# Innovative Modifications to the LASSI‐L Cognitive Challenge Test

**DOI:** 10.1002/alz70857_098431

**Published:** 2025-12-24

**Authors:** Alexandra Ortega

**Affiliations:** ^1^ University of Miami Leonard M. Miller School of Medicine, Miami, FL, USA

## Abstract

**Background:**

Early detection of Alzheimer's disease (AD) and related dementias (ADRD) is critical as this is when emerging therapies are most effective. Traditional cognitive assessments often lack sensitivity to detect subtle early deficits, and specificity required to identify ADRD. To address these limitations, our lab has developed and continuously refined the Loewenstein‐Acevedo Scales for Semantic Interference and Learning (LASSI‐L), a novel cognitive challenge test (CCT) that targets cognitive vulnerabilities associated with AD.

**Method:**

Core components include the assessment of proactive semantic interference (PSI) and the failure to recover from proactive semantic interference (frPSI), aspects associated with deficits in semantic processing. The LASSI‐L employs semantic category cues during encoding and retrieval to facilitate learning, reduce reliance on individualized learning strategies and enhance recall. Early executive and inhibitory control deficits can be assessed using the Percent Intrusion Errors (PIE) metric. This metric represents the ratio of semantic intrusion errors (SIEs), which occur when individuals fail to suppress irrelevant, competing information in favor of identifying correct targets. The addition of a delayed semantic source memory recognition task was designed to evaluate deficits in source memory and enhance detection of SIEs. This CCT has been fully digitized featuring a Cloud‐based software using voice recognition technology and is available in English and Spanish. Integration with amyloid PET imaging and plasma‐based biomarkers (e.g., *p*‐tau181, *p*‐tau217), to improve diagnostic precision and validate its use in identifying AD‐specific cognitive deficits.

**Results:**

Delayed semantic source memory recognition task resulted in increased identification of SIEs linked to executive function and source memory deficits. SIEs have been found to be highly discriminative between those with amnestic Mild Cognitive Impairment (aMCI) with amyloid PET positivity. SIEs also differentiate between aMCI who are plasma *p*‐tau181+ versus *p*‐tau181‐and those who are *p*‐tau217+ versus *p*‐tau217‐. Deficits have been correlated with amyloid load and hippocampal atrophy. PIE has shown high sensitivity and specificity, distinguishing preclinical AD from cognitively unimpaired individuals.

**Conclusion:**

This presentation will discuss the innovative modifications to the LASSI‐L. This CCT holds promise as an accessible tool for early AD/ADRD detection, particularly in diverse and underserved populations, and complements emerging biomarker‐based approaches to diagnosis.

